# Translation, cross-cultural adaptation, and validation of the Italian version of the anterior cruciate ligament–return to sport after injury (ACL-RSI) scale and its integration into the K-STARTS test

**DOI:** 10.1186/s10195-021-00622-7

**Published:** 2022-02-21

**Authors:** Edoardo Monaco, Gabriele Pisanu, Alessandro Carrozzo, Alessandro Giuliani, Jacopo Conteduca, Matteo Oliviero, Luca Ceroni, Bertrand Sonnery-Cottet, Andrea Ferretti

**Affiliations:** 1 Orthopaedic and Trauma Surgery Unit, AOU Sant’Andrea, La Sapienza University of Rome, Via di Grottarossa 1035-1038, 00189 Rome, Italy; 2 Orthopaedic and Trauma Surgery Unit, AO Santa Croce e Carle, Via Michele Coppino, 26, 12100 Cuneo, Italy; 3 Orthopaedic and Trauma Surgery Unit, Fondazione Policlinico Universitario Agostino Gemelli IRCCS, Via della Pineta Sacchetti, 217, 00168 Rome, Italy; 4 Orthopaedic and Trauma Surgery Unit, AO Vito Fazzi, Piazzetta Muratore, 73100 Lecce, Italy; 5 Centre Orthopédique Santy, Ramsay Générale de Santé, 24 Avenue Paul Santy, 69008 Lyon, France; 6 Orthopaedic and Trauma Surgery Unit, University of Turin, Via Giuseppe Verdi, 8, 10124 Turin, Italy; 7 Psychological Sciences and Techniques, Psychology Department, University of Turin, Via Giuseppe Verdi, 8, 10124 Turin, Italy

**Keywords:** ACL, ACL-RSI, K-STARTS, Italian translation, Return to sport, Return to play

## Abstract

**Background:**

The timing of a return to sport (RTS) after anterior cruciate ligament reconstruction (ACLR) represents a major subject of debate in sports medicine practice. Recently, the Knee Santy Athletic Return to Sport (K-STARTS) composite test was validated. This consists of a battery of physical tests and a psychological evaluation using the anterior cruciate ligament–return to sport after injury scale (ACL-RSI). This study aimed to translate the ACL-RSI and K-STARTS from English to Italian and determine the scale’s reliability and validity in an Italian context.

**Methods:**

The translation and cultural adaptation process was performed according to the guidelines for the cross-cultural adaptation of self-report measures. The patients were asked to fill an anonymized online form created for this purpose that included the KOOS, the Lysholm, the IKDC-SKF, and the Italian translation of the ACL-RSI (ACL-RSI-It). After 1 week, the attendees were asked to repeat the ACL-RSI-It to investigate the test–retest reliability.

**Results:**

The final study population comprised 115 patients who underwent ACLR, with a mean follow-up of 37.37 ± 26.56 months. The ACL-RSI-It showed axcellent internal consistency (Cronbach’s *α* = 0.963), reliability (test–retest ICC = 0.966), and good construct validity (positive correlations with the other scales were above 75%).

**Conclusions:**

The ACL-RSI-It is valid, reliable, and comparable to the original English version of the questionnaire for Italian-speaking patients. It can be used to assess the psychological readiness of patients for a RTS after primary and unilateral ACLR, and can be integrated into the Italian K-STARTS test.

**Level of evidence:**

Level II.

**Supplementary Information:**

The online version contains supplementary material available at 10.1186/s10195-021-00622-7.

## Introduction

Anterior cruciate ligament reconstruction (ACLR) represents one of the most commonly performed orthopedic surgical procedures [1–3].

Several tools have been developed to broadly assess functional outcomes and to assess overall function after knee injuries [4–7]. However, the ability of each tool to determine whether a patient can return to sport (RTS) has not been objectively validated [8–10].

Historically, the primary clinical target of RTS was the ability to achieve similar side-to-side results at physical performance tests [9, 11]. Even though good physical performance outcomes are frequently reported after ACLR, a lower proportion of patients—especially competitive athletes—return to their preinjury sports [12]. Only 65% of patients return to their preinjury level in their sport and only 55% return to competitive sport [13]. Due to the mismatch between the RTS rate and physical performance outcomes after ACLR, the focus has moved on to psychological factors, such as a fear of re-injury, a lack of confidence, and kinesiophobia [14–19].

Concerning the psychological impact on RTS after ACL injury or reconstruction, in 2008, Webster et al. [20] developed the ACL–return to sport after injury (ACL-RSI) scale in the English language. This scale comprises 12 questions that evaluate emotions, confidence in performance, and risk appraisal of athletes after ACL injuries. The ACL-RSI has a strong predictive value for RTS [20, 21] and has shown good construct validity compared with other subjective scores.

In 2018, to merge the evaluation of physical performance with that of psychological factors when assessing fitness for RTS after ACL reconstruction, the Knee Santy Athletic Return to Sport (K-STARTS) composite test was validated [22]. This consists of a battery of tests that aim for an objective outcome measure of functional improvement after ACL reconstruction, and it is calculated as the sum of eight components: the ACL-RSI results and seven physical tests [22]. It has a high completion rate, high reproducibility, and a high sensitivity to change. Based on these findings, the K-STARTS test has been considered an appropriate and objective outcome measure for functional improvement after ACL reconstruction [23].

Therefore, the validation of an Italian version of the ACL-RSI and its integration into a translated version of the K-STARTS test could help with the evaluation of both the psychological and the physical factors that affect RTS in the Italian-speaking population.

This study aimed to translate the ACL-RSI and K-STARTS from English to Italian and to determine the scale’s reliability and validity in an Italian context. We hypothesized that an Italian version of the ACL-RSI scale would have good construct validity compared to the IKDC-SKF, Lysholm, and KOOS. Therefore, it could demonstrate substantial reliability as a patient-reported outcome for athletes who undergo ACL reconstruction, and could be integrated into an Italian version of K-STARTS.

## Methods

### Translation and cross‑cultural adaptation

The translation and cultural adaptation process was performed according to the guidelines for the cross-cultural adaptation of self-report measures by Beaton et al. [24]. The final version was validated as stated in the Consensus-Based Standards for the Selection of Health Status Measurement Instruments (COSMIN) guidelines [25, 26].

The original English version was independently translated into Italian by one orthopedic surgeon with a good knowledge of English and an English teacher without a medical background; both were native Italian speakers. The initial Italian version of the questionnaire was chosen after a consensus meeting where discrepancies between the two initial Italian versions were debated and corrected. At the back translation stage, two native English speakers with fluent Italian, who were not informed of the study’s purpose and were blinded to the original English version, were asked to translate the chosen Italian version into English. An expert committee composed of the four translators, another orthopedic surgeon, and a psychometrist compared the original English versions of the KSTARS and ACL-RSI scale with the back-translated versions of the tests. A prefinal version was created and tested on 10 Italian-speaking volunteers to determine their comprehension of the Italian ACL-RSI version (ACL-RSI-It). None of the volunteers reported interpretation problems or redundancy. The authors then approved the final versions (see Additional files 1 and 2).

### Population

Institutional Review Board approval was granted for this study. All participants and their parents gave valid consent to participate. A retrospective analysis of prospectively collected data for consecutive patients who underwent ACLR at the “Kilk Kilgour” Sports Traumatology Center, Sant’Andrea University Hospital of Rome, or the orthopedic surgery department of Santa Croce e Carle of Cuneo between January 1st, 2018 and January 1st, 2020 was conducted. The inclusion criteria were: (1) primary ACLR; (2) ≥ 18 years old; and (3) ≥ 6 months of follow-up. Participants were excluded if they:(1) underwent a bilateral ACLR, (2) had a history of previous knee injuries, (3) suffered from multi-ligament knee injuries or a concomitant fracture other than Segond’s, (4) had radiographic findings of knee osteoarthritis, or (5) did not have a good understanding of both the Italian and English languages.

In order to minimize the bias related to the multicenter design of this study, the patients all performed the same postoperative rehabilitation protocol. This included a brace locked in extension for the first 4 weeks, with weight bearing allowed with crutches from the first postoperative day; recovery of knee range of motion (ROM) starting at 2 weeks, with the brace temporarily removed during exercises; at 4 weeks, patients were allowed to walk without crutches and the brace; progressively increased ROM and muscle strengthening until the third month; from the third to the sixth month, patients underwent a progressive muscle strengthening program, and sport, specific exercises were performed. A return to sporting activities was allowed at 6 postoperative months.

After a preliminary telephone call explaining the study’s purpose, the patients were asked to fill an anonymized online form created for this purpose and administered via Google Forms (Google LLC, Mountain View, CA, USA). The form consisted of a first section asking for demographic data and a second section that included the KOOS, the Lysholm, the IKDC-SKF, and the ACL-RSI-It [27–29]. After 1 week, the attendees were asked to repeat the ACL-RSI-It to investigate the test–retest reliability.

### Patient‑reported outcome measures

The original ACL-RSI scale is a self-administered questionnaire that consists of 12 items evaluated with a 10-cm visual analog scale (VAS) from 0 to 100 in 10-point increments. It is subdivided into three aspects that cover (1) emotions (five questions), (2) confidence in one’s performance (five questions), and (3) risk appraisal (two questions) [20]. Later, a modified ACL-RSI was developed by the same team; in this, the VAS scales with 11-point Likert scales in boxes with 10-point increments from 0 to 100 [18]. The total score is calculated by adding the values of the 12 items and then taking the percentage of the amount. High scores are indicative of a positive psychological response.

The IKDC-SKF [29], the KOOS [28], and the Lysholm score [27] are scales that are widely utilized in clinical practice; these were the references for the current study in their validated Italian versions.

### K-STARTS score

The patient can perform the K-STARTS test if no deficits are present above 40% relative to the contralateral limb at a prior executed isokinetic test. The K-STARTS test provides a psychological status and physical performance assessment and is composed of four sections.

The first section of the K-STARTS appraises psychological readiness for RTS and is determined by the outcome of the ACL-RSI questionnaire. Three K-STARTS points are given for ACL-RSI scores of 76% or higher, 2 points for scores between 64 and 75%, 1 point for scores between 56 and 63%, and 0 points for scores less than 55%.

The second section of the K-STARTS evaluates neuromuscular control, as determined by the outcome of the Qualitative Analysis of Single-Leg Squat (QASLS). This tool provides an analysis of movement occurring in the arms, trunk, pelvis, thighs, knees, and feet during single-leg loading tasks [30]. The QASLS score ranges between 0 (best) and 10 (worst), with points increasing for inappropriate movement strategies. A patient with a QASLS of 0, 1, 2, or ≥ 3 scores, respectively, 3, 2, 1, and 0 points in K-STARTS. Furthermore, 3 points are deducted if a patient is judged to have a dynamic valgus of the limb during the single-leg loading task.

The third section of the K-STARTS is an estimation of the limb symmetry index. Four categories of hop test (single, triple, side, and crossover) are carried out, and the percentage deficit of the distance hopped on the involved leg compared to that hopped on the uninvolved contralateral leg is computed [23, 31, 32]. For each of the hop tests, a limb symmetry index of 90% or more corresponds to 3 K-STARTS points, between 80 and 89% corresponds to 2 points, and 79% or less to 1 point; if pain occurs during the test, no points are attributed.

The fourth section evaluates the ability to change direction using the Modified Illinois Change of Direction Test (MICODT) [33]. An average MICODT time of ≤ 12.5 s scores 3 K-STARTS points. If the time ranges from 12.51 to 13.5 s, it scores 2 points, and if the time is > 13.5 s, it scores 1 point. Moreover, if pain prevents the test, no points are given.

The total score is calculated as a percentage. If the final score is less than 50 points, a return to training is discouraged. If the score ranges between and 50 and 65, noncontact nonpivot training is allowed. For a score ranging between 65 and 80, noncontact pivot training is allowed. If the score is higher than 80, the athlete is allowed to return to contact training activity.

### Statistical analysis

Statistical analysis was performed with IBM SPSS version 25.0 for macOS (IBM, Armonk, NY, USA). The significance threshold was set at *p* < 0.05.

Descriptive data were presented in the form of mean ± standard deviation and percentage.

Floor or ceiling effects were considered present if the proportion of patients (%) who had the minimum (0) or maximum (100) score on the scale was more than 15% [34].

Internal consistency was estimated using Cronbach’s *α* coefficient. Homogeneity between items within a questionnaire was considered excellent for *α* > 0.90 [34].

The intraclass correlation coefficient (ICC) with a 95% confidence interval was calculated to assess the instrument’s stability over time. To determine test–retest reliability, we used Spearman’s correlation coefficient and the ICC* q*; reproducibility was considered either excellent (*q* > 0.75), good (0.75 < *q* < 0.40), or poor (*q* < 0.40) [35]. A value of greater than 0.40 was considered acceptable.

The construct validity was assessed by comparing the ACL-RSI-It with the subjective IKDC, the different components of the KOOS, and the Lysholm score using Spearman’s correlation coefficient; the correlation was considered strong (*r* ≥ 0.5), medium (0.5 > *r* > 0.3), or small (*r* < 0.3).

Discriminant validity was tested by a Mann–Whitney U test between patients who returned to their previous level in sport and those who could not return to the same level.

## Results

### Cross‑cultural adaptation

Neither the Italian translation nor the subsequent English back translation of the ACL-RSI scale or the K-STARTS test created any major linguistic problems. The pilot ACL-RSI-It was completed without questions or doubt from the patients.

### Study participants

**Fig. 1:**
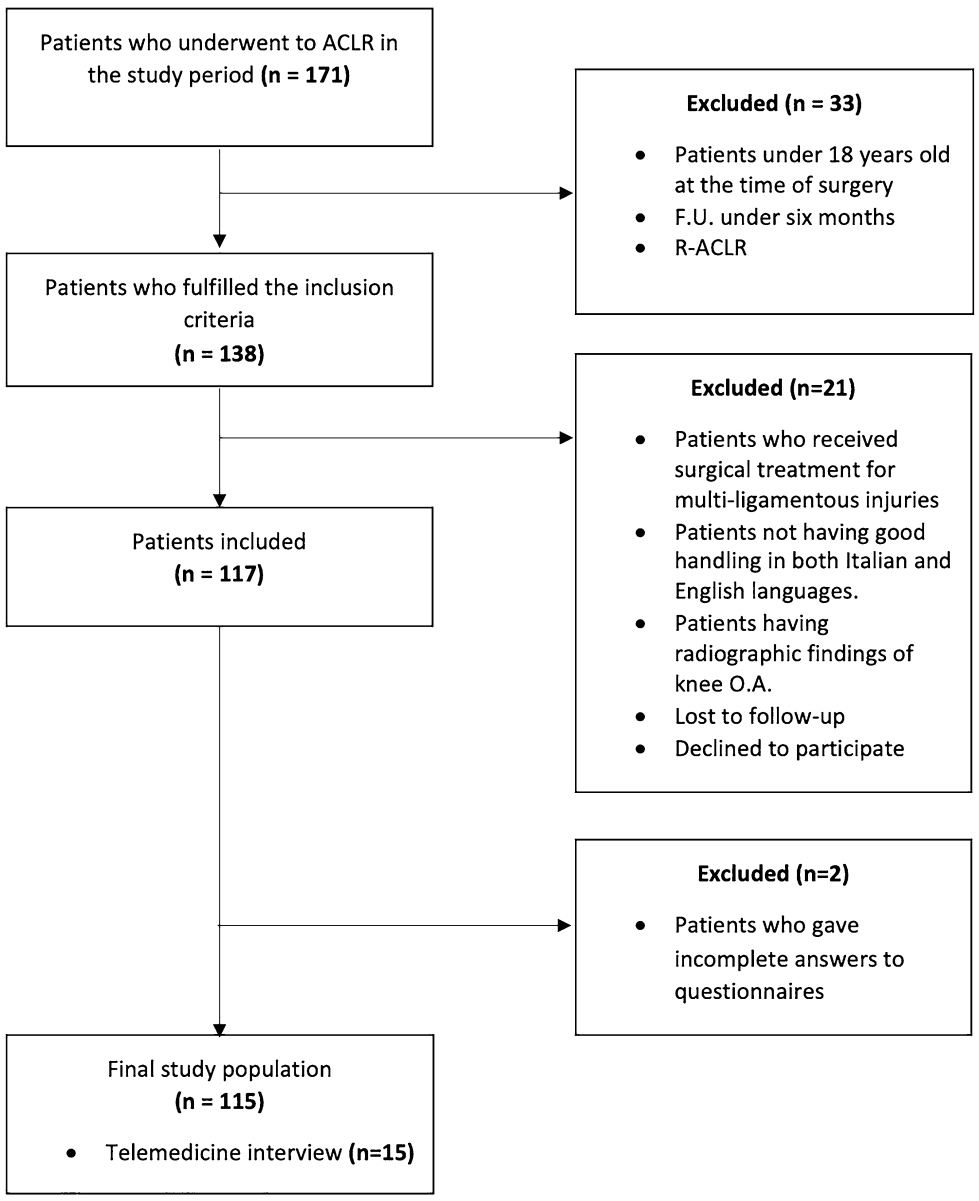
Flowchart of the study, created in line with the Strengthening the Reporting of Observational Studies in Epidemiology (STROBE) statement (http://www.strobestatement.org). *ACLR* anterior cruciate ligament reconstruction, *F.U.* follow-up, *O.A.* osteoarthritis, *R-ACLR* revision anterior cruciate ligament reconstruction

Overall, 171 patients underwent ACLR during the study period at the two institutions, and 138 met the inclusion criteria. Seven patients (5.07%) were lost to follow-up, three patients (2.17%) declined to participate, and two patients (1.45%) provided incomplete answers on their questionnaires. The study flow is presented in Fig. 1. The final study population comprised 115 patients with a mean follow-up of 37.37 ± 26.56 months. The demographics of the study population are summarized in Table 1.

**Table 1:** Patient demographics

	All patients (*N* = 115)
Age, mean ± SD, (range), years	25.6 ± 8.2 (18.1–58.6)
Sex,* n* (%)	
Male	83 (72.2)
Female	32 (27.8)
Preinjury activity level, *n* (%)	
Professional athletes	16 (13.9)
Competitive sports activity	14 (12.2)
Recreational sports activity at least three times a week	30 (26.1)
Occasional recreational sports activity	55 (47.8)
Follow-up, mean ± SD (range), months	37.37 ± 26.56 (6–75)
Return to preinjury activity level, *n* (%)	92 (80)

### Internal consistency

The internal consistency of the ACL-RSI-It scale based on the strength of the correlation among the 12 items was excellent, with a Cronbach’s* α* of 0.963.

### Reliability

The reproducibility of the scale was analyzed by the test–retest. The mean ACL-RSI-It score was 85 ± 26.9 and 86.6 ± 28 at the first and second administrations. The ICCs were greater than 0.9 for the total and for three aspects of the ACL-RSI-It (ICC = 0.966, 95% CI; 0.950–0.976), indicating that the test–retest reliability of the ACL-RSI-It was excellent. The correlation between the two versions was strong, with a Spearman’s correlation coefficient of *r* = 0.911 (*p  *< 0.001). Reproducibility was excellent, with an ICC *q* = 0.981 [0.976–0.986] (*p  *< 0.001).

### Description of the results and feasibility

The ACL-RSI-It scale was not normally distributed according to the Kolmogorov–Smirnov test (*p* < 0.001). None of the items had a poor corrected item–total correlation, indicating that all items should be included in the ACL-RSI-It. The floor effect, corresponding to the percentage of patients with a score of 0 for each question, varied between 1.7% and 6.1%. The ceiling effect, corresponding to the percentage of patients with a score of 10 for each question, varied between 3.5% and 50.4%.

### Construct validity

The principal component analysis showed one underlying factor of the ACL-RSI-It with an explained variance of 72.43% and an eigenvalue of 1. Significant positive correlations between ACL-RSI-It and all the reference scales were present (*p* < 0.05, Table 2).

**Table 2:** Correlations between the Italian ACL-RSI scale and the IKDC-SKF, Lysholm score, and KOOS subscales

	Median (IQR)	Correlation	*p*-value
IKDC-SKF	71.26 (9.19)	0.41	**< 0.05**
Lysholm score	93 (11.5)	0.55	**< 0.05**
KOOS Symptoms/Stiffness	89.20 (14.29)	0.44	**< 0.05**
KOOS Pain	70.59 (14.71)	0.51	**< 0.05**
KOOS ADL	75 (2.2)	0.41	**< 0.05**
KOOS Sport/Rec	60 (20)	0.50	**< 0.05**
KOOS QoL	62.5 (31.25)	0.63	**< 0.05**

Bold values indicate statistical significance

### Discriminant validity

The discriminant validity of ACL-RSI-It was demonstrated to be good. The subgroup of patients who returned to sport at the same or higher level had a mean result of 96 ± 15, and those who could not RTS had a mean result of 52.3 ± 27.5 (Mann–Whitney *U* = 145).

## Discussion

This study’s main finding was that the current translated version of the ACL-RSI scale could be validated and integrated into the K-STARTS composite test to assess readiness to return to sport. The K-STARTS test combines physical functional tests with a psychological evaluation through the ACL-RSI score. Recently, interest in the psychological implications of returning to sport after ACLR have grown [15]. Fear of re-injury, self-confidence, and motivation are factors that are as important as the surgery and rehabilitation protocol in the ability to return to preinjury sport [16, 36, 37]. This new implication of the psychological aspect could explain the mismatch between satisfactory physical performance outcomes and a failure to RTS or a lower level of RTS. Therefore, the original version of ACL-RSI was developed to specifically assess the psychological impact of a RTS after ACLR [20].

The ACL-RSI-It showed excellent internal consistency (Cronbach’s *α* = 0.963) based on the correlation among the 12 items considered, and its* α* value was comparable to those of the other versions of the ACL-RSI in other languages: English (0.96) [20], Swedish (0.95) [38], French (0.96) [39], Dutch (0.94) [40], Turkish (0.86) [17], Chinese (0.94) [41], Lithuanian (0.94) [42], Spanish (0.9) [43], Japanese (0.91) [44], and Norwegian (0.95) [45]. Another Italian group validated an Italian version of the ACL-RSI during the period of the present study. Although the translation of the questions differed slightly, we found similar results in terms of internal consistency (Cronbach *α* = 0.953) and the other measurement properties [46]. Test–retest reliability for the ACL-RSI-It was also excellent and comparable with previous studies [17, 38–45]. According to previous studies [17, 20, 38, 39, 41, 45], the discriminant validity of the ACL-RSI-It score is proven by the significantly higher results in patients who returned to sport compared to those who did not. There was a significant difference in ACL-RSI-It scores between the two groups. The ACL-RSI-It scale showed excellent reliability in repeated measures (ICC = 0.966; 95% CI 0.950–0.976); similar results were reported from other cross-cultural validations [39–41, 44, 45].

The construct validity of the ACL-RSI-It scale was analyzed by testing its correlations with three knee-specific reference scales that were previously validated in Italian and are widely used among orthopedic surgeons [27–29]. The ACL-RSI-It had positive correlations of > 75% with the IKDC-SKF, Lysholm, and subscales of KOOS scores. Thus, the ACL-RSI-It has good construct validity according to the COSMIN guidelines [34].

The IKDC-SKF was developed to measure symptoms and limitations in daily activities and sports, and to only partially evaluate psychological factors related to returning to sports. The correlation between the ACL-RSI-It and IKDC-SKF was medium (*r* = 0.41, *p* < 0.01); this result is similar to those seen for previously published translations into other languages [17, 39–42, 44, 45].

Medium correlations were observed between the ACL-RSI-It scale and the KOOS subscales Symptoms (S; *r* = 0.44) and Function in Daily Life (ADL; *r* = 0.41). On the other hand, the KOOS subscales Function in Sport and Recreational Activities (Sport/Rec; *r* = 0.5), Pain (P; *r* = 0.5), and Knee-Related Quality of Life (QoL; *r* = 0.63) were strongly correlated to the ACL-RSI-It scale. As noted in previous studies, the ACL-RSI-It scale was most strongly correlated with the KOOS QoL subscale among all the subscales [17, 38, 39, 41, 43, 45]. The authors agree with our French colleagues who hypothesized that a good quality of life is strongly associated with returning to sports after ACLR among active sports populations [17, 38–41, 43–45].

The Lysholm score was developed to evaluate the patient’s functional status after ACLR, and an increase in this score over time is known to occur [47, 48]. In our study, a strong correlation was also found between the ACL-RSI-It and the Lysholm score (*r* = 0.55), and this correlation was similar to or higher than that seen in other studies [17, 39, 41, 44].

One of the strengths of this work is that it has been possible to make the use of a tool such as the K-STARTS available to native-Italian-speaking professionals. The composite test includes the version of the ACL-RSI translated and validated by us. This increases the clinical relevance of the ACL-RSI, as its outcome significantly contributes to the score obtained in the K-STARTS and gives an indication of patient’s readiness for the RTS. Another strength of the current study is the multicenter collection of patients who practiced sports at various levels. A limitation of the current investigation is the presence of a ceiling effect due to the mean ACL-RSI-It score of 85, which is higher than seen in previous similar studies [17, 38–45]. The authors attribute this ceiling effect to the recruitment of patients with more than 12 months of follow-up; therefore, the results could have stabilized at higher scores. Moreover, some patients with unsuccessful results declined the invitation to participate in the questionnaire, removing possible lower scores.

This translated version of the ACL-RSI has satisfactory internal consistency, excellent test–retest reliability, and notable construct validity and discriminant validity. The ACL-RSI-It is valid, reliable, and comparable to the original English version of the questionnaire for Italian-speaking patients. It can be used to assess psychological readiness for a RTS in patients after primary and unilateral ACLR and when performing the K-STARTS test.

## Supplementary Information

**Additional file 1.** ACL-RSI-It.

**Additional file 2.** Italian version of KSTARTS.

## Data Availability

The database generated during and/or analyzed during the current study is not publicly available but is available from the corresponding author on reasonable request.

## Abbreviations

ACL-RSI-It: Italian version of the ACL-RSI
ACL-RSI: ACL–return to sport after injury scale
ACLR: Anterior cruciate ligament reconstruction
ADL: Activity in daily life
COSMIN: Consensus-Based Standards for the Selection of Health Status Measurement Instruments
I-PRRS: Injury-Psychological Readiness to Return to Sport scale
ICC: Intraclass correlation coefficient
IKDC-SKF: International Knee Documentation Committee Subjective Knee Form
K-STARTS: Knee Santy Athletic Return to Sport
KOOS: Knee Injury and Osteoarthritis Outcome Score
MICODT: Modified Illinois Change of Direction Test
OA: Osteoarthritis
QASLS: Qualitative Analysis of Single-Leg Squat
QOL: Quality of life
RTS: Return to sport
STROBE: Strengthening the Reporting of Observational Studies in Epidemiology
TSK: Tampa Scale for Kinesiophobia
VAS: Visual analog scale
