# Supplementary material for: Translation, cross-cultural adaptation, and validation of the Italian version of the anterior cruciate ligament–return to sport after injury (ACL-RSI) scale and its integration into the K-STARTS test
Source: J Orthop Traumatol. 2022 Feb 21;23:11. doi: 10.1186/s10195-021-00622-7 (PMC8861218; doi:10.1186/s10195-021-00622-7)
Supplement: Supplementary file 1 — Additional file 1. ACL-RSI-It. [file 10195_2021_622_MOESM1_ESM.docx]

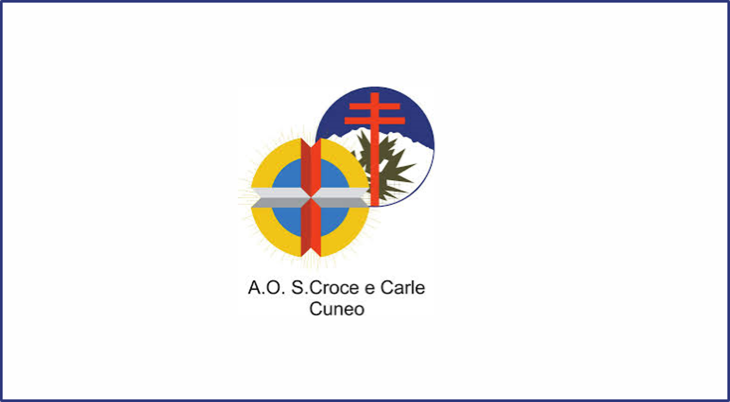


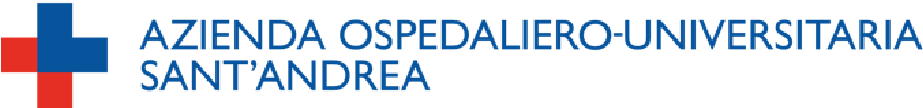


| **QUESTIONARIO ACL-RSI PER LA VALUTAZIONE DEL RITORNO ALLO SPORT DOPO LESIONE DEL LEGAMENTO CROCIATO ANTERIORE**  Istruzioni:  Si prega di rispondere alle seguenti domande riferendosi allo sport principale prima dell'infortunio. Per ogni domanda, spunti un numero tra le due estremità per indicare come si sente in questo momento. | | |
| --- | --- | --- |
| È fiducioso di poter praticare il suo sport allo stesso livello precedente all'infortunio? | | |
| Per niente fiducioso/a | 0 1 2 3 4 5 6 7 8 9 10 | Totalmente fiducioso/a |
| Ritiene che sia probabile infortunarsi nuovamente al ginocchio praticando il suo sport? | | |
| Estremamente probabile | 0 1 2 3 4 5 6 7 8 9 10 | Per niente probabile |
| Si sente nervoso all'idea di praticare il suo sport? | | |
| Estremamente nervoso/a | 0 1 2 3 4 5 6 7 8 9 10 | Per niente nervoso/a |
| Si ritiene fiducioso che il suo ginocchio non cederà praticando il suo sport? | | |
| Per niente fiducioso/a | 0 1 2 3 4 5 6 7 8 9 10 | Totalmente fiducioso/a |
| Si ritiene fiducioso di poter praticare il suo sport senza preoccuparsi del ginocchio? | | |
| Per niente fiducioso/a | 0 1 2 3 4 5 6 7 8 9 10 | Totalmente fiducioso/a |
| Ritiene frustrante di dover prendere in considerazione il suo ginocchio mentre pratica il suo sport? | | |
| Estremamente frustrante | 0 1 2 3 4 5 6 7 8 9 10 | Per niente frustrante |
| Ha paura di re-infortunarsi il ginocchio facendo sport? | | |
| Paura Estrema | 0 1 2 3 4 5 6 7 8 9 10 | Nessuna paura |
| È fiducioso che il suo ginocchio regga sotto pressione? | | |
| Per niente fiducioso/a | 0 1 2 3 4 5 6 7 8 9 10 | Totalmente fiducioso/a |
| È preoccupato di infortunarsi accidentalmente il suo ginocchio facendo sport? | | |
| Estremamente preoccupato | 0 1 2 3 4 5 6 7 8 9 10 | Per niente preoccupato |
| Il pensiero di dover eventualmente ripetere l’intervento chirurgico e il processo riabilitativo le impedisce di praticare sport? | | |
| Tutte le volte | 0 1 2 3 4 5 6 7 8 9 10 | Nessuna volta |
| È fiducioso nelle sue capacità di poter far bene nel suo sport? | | |
| Per niente fiducioso/a | 0 1 2 3 4 5 6 7 8 9 10 | Totalmente fiducioso/a |
| Si sente tranquillo nel praticare il suo sport? | | |
| Per niente tranquillo/a | 0 1 2 3 4 5 6 7 8 9 10 | Totalmente tranquillo/a |
