# Supplementary material for: Translation, cross-cultural adaptation, and validation of the Italian version of the anterior cruciate ligament–return to sport after injury (ACL-RSI) scale and its integration into the K-STARTS test
Source: J Orthop Traumatol. 2022 Feb 21;23:11. doi: 10.1186/s10195-021-00622-7 (PMC8861218; doi:10.1186/s10195-021-00622-7)

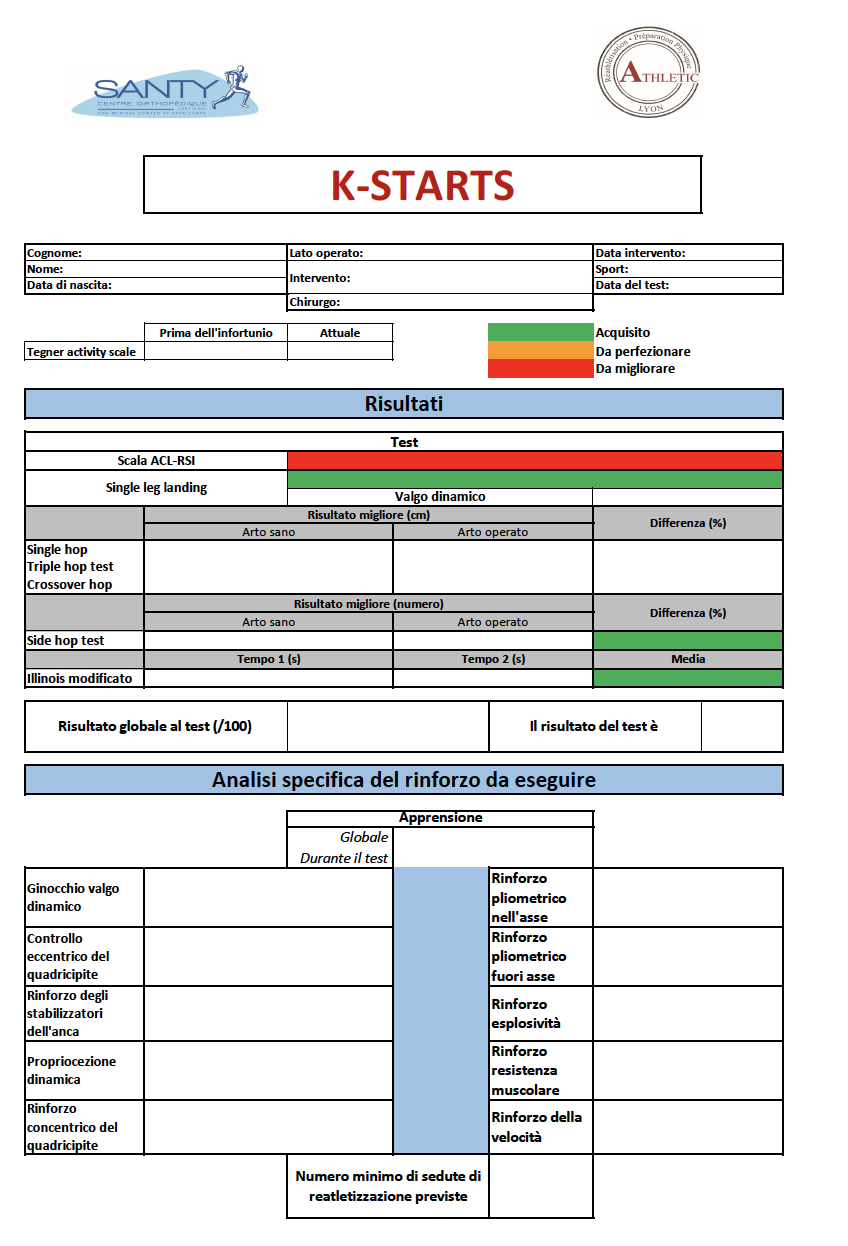


**Legenda**

Valgo dinamico: Sì/No

Apprensione globale: No/Alla ripresa dell’attività/Alla ripresa della competizione

Apprensione durante il test: Sì/No

Ginocchio valgo dinamico: No/Destro/Sinistro/Bilaterale

Controllo eccentrico del quadricipite: Acquisito/Destro/Sinistro/Bilaterale

Rinforzo degli stabilizzatori dell’anca: Acquisito/Destro/Sinistro/Bilaterale

Propriocezione dinamica: Acquisita/Destra/Sinistra/Bilaterale

Rinforzo concentrico del quadricipite: Acquisito/Destro/Sinistro/Bilaterale

Rinforzo pliometrico nell’asse: Acquisito/Destro/Sinistro/Bilaterale

Rinforzo pliometrico fuori asse: Acquisito/Destro/Sinistro/Bilaterale

Rinforzo esplosività: Acquisito/Destro/Sinistro/Bilaterale

Rinforzo resistenza muscolare: Acquisito/Destro/Sinistro/Bilaterale

Rinforzo della velocità: Acquisito/Da migliorare/Da perfezionare


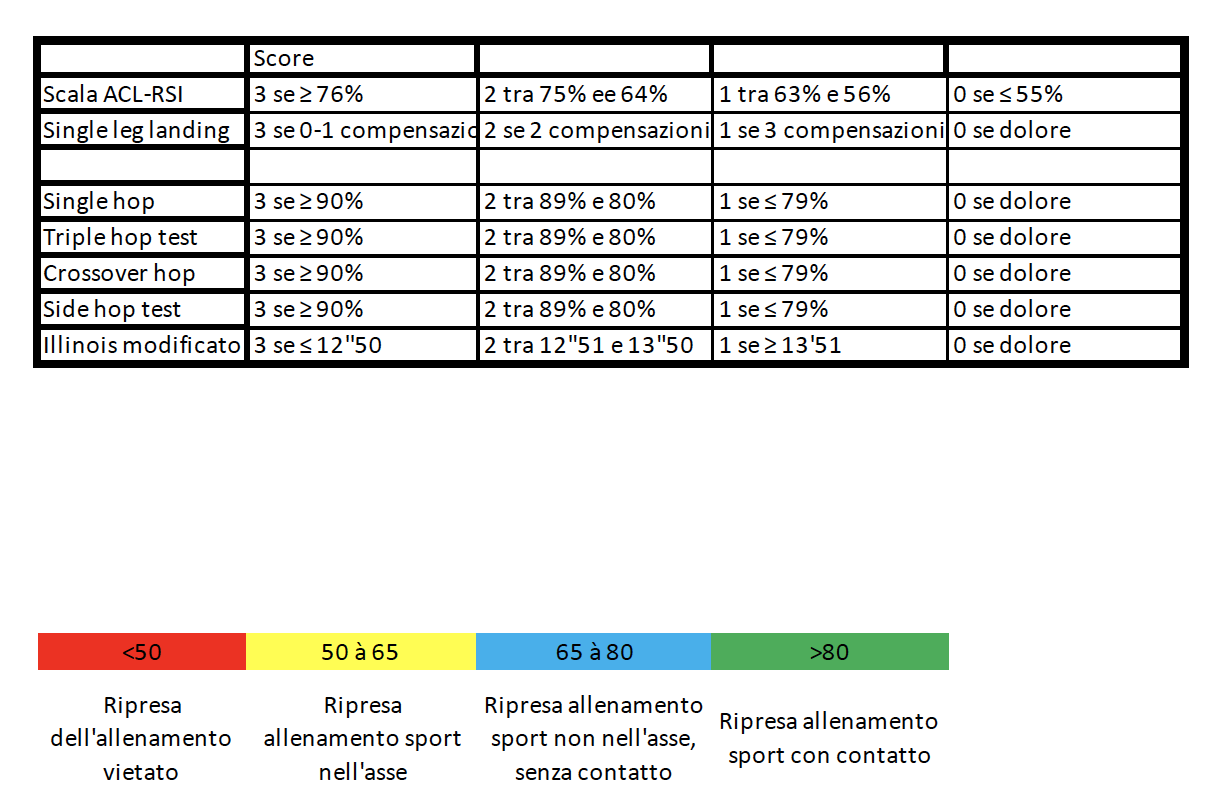

Supplement: Supplementary file 2 — Additional file 2. Italian version of KSTARTS. [file 10195_2021_622_MOESM2_ESM.docx]
